# Supplementary material for: Oncogene inference optimization using constraint-based modelling incorporated with protein expression in normal and tumour tissues
Source: R Soc Open Sci. 2020 Mar 18;7(3):191241. doi: 10.1098/rsos.191241 (PMC7137941; doi:10.1098/rsos.191241)

Supplementary material S1

Contents:

1. Choke-Point Metabolites
2. Flux-Sum Synthesis Rate
3. Flux Patterns for Normal, Cancer and Mutants
4. Logarithmic Fold Change Ratio for the Template and Mutants
5. Similarity Indicator and Similarity Ratio
6. Nested Hybrid Differential Evolution (NHDE)
7. Categories of Flux Variances Between the Normal and Mutant Models

1. Choke-Point Metabolites

- Suppose a genome-scale metabolic network as the below pathways:

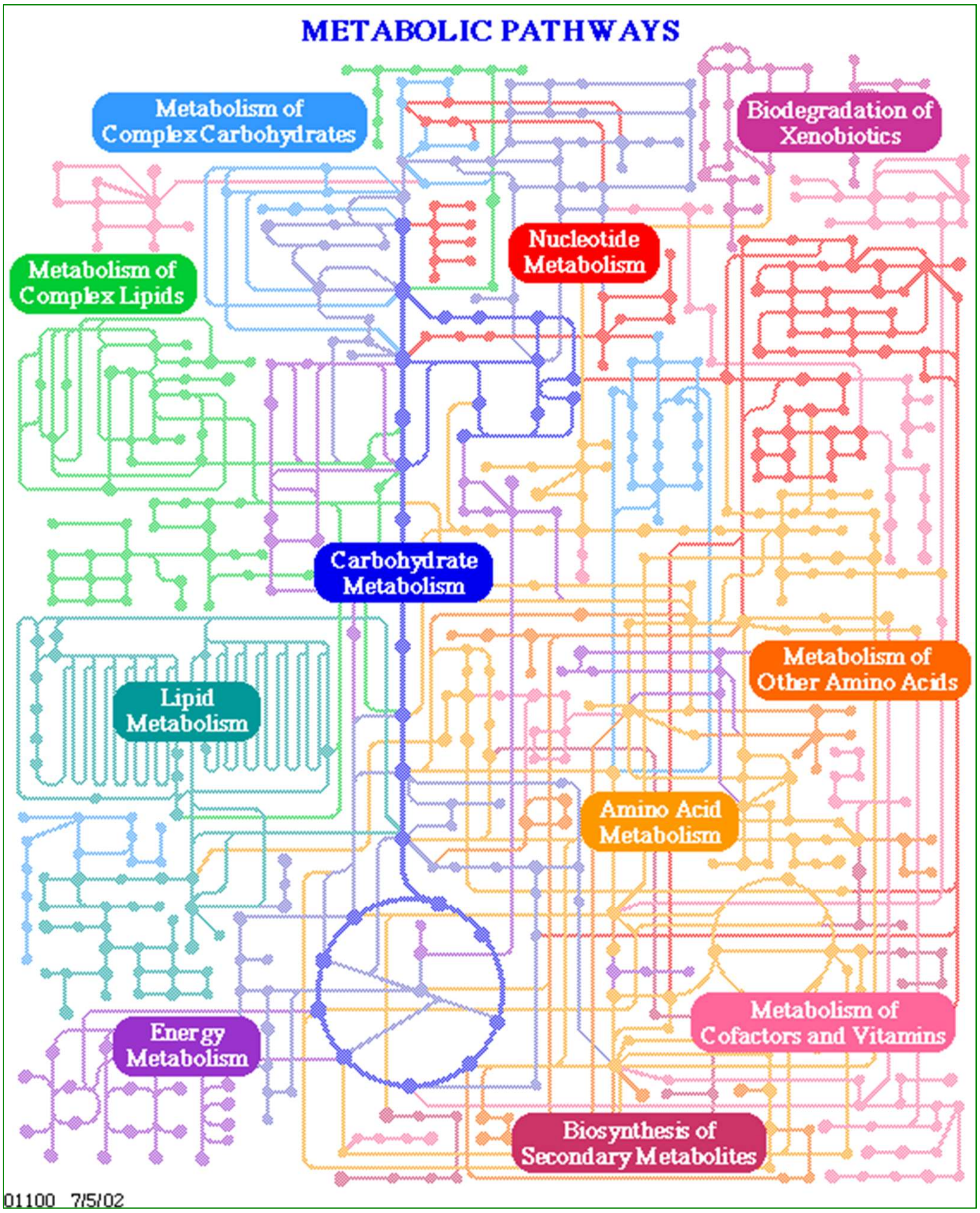

- Each node in the network indicates a metabolite, and metabolic reactions are connected with nodes as represented by lines
- Consider the metabolic network at steady-state, it can be formulated as

$$\mathbf{Nv} = \mathbf{0}$$

- From the genome-scale metabolic model, we can apply topology analysis for the stoichiometric matrix, **N**, to discriminate choke-point metabolites.
- Three categories of choke-point metabolites are defined as follows:
- A choke-point metabolite connected with a single-ingoing and multi-outgoing reaction:

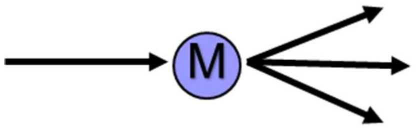

- A choke-point metabolite connected with a multi-ingoing and single-outgoing reaction:

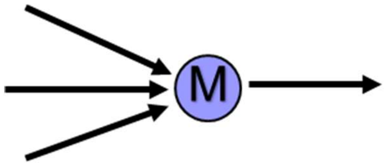

- A choke-point metabolite connected with a single-ingoing and single-outgoing reaction:

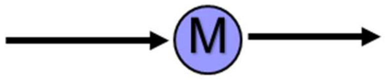

2. Flux-Sum Synthesis Rate

- Suppose that a metabolic network consisted of the metabolite, M1, which participates in cytoplasm (c) and mitochondrion (m).
- Five reversible reactions are linked to the metabolites, M1_c and M1_m, and the flux distributions are shown in the below figure. v_{fi} denotes as the forward fluxes, and v_{bi} are backward fluxes. The stoichiometric coefficients for the forward reaction are assumed to be 1, and -1 for the backward reactions.

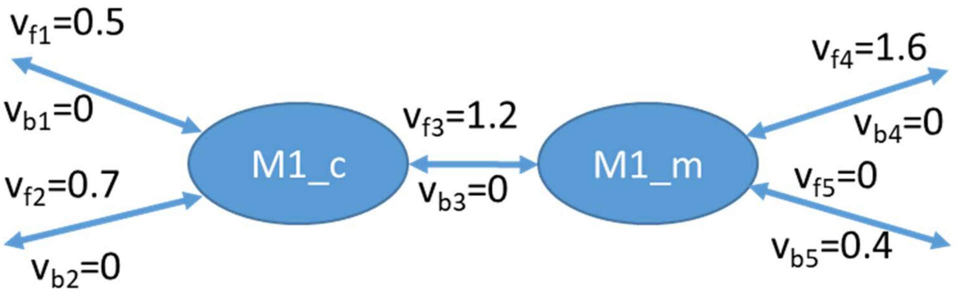

- Flux-sum synthesis rates for the metabolite, M1, in cytoplasm and mitochondrion are respectively obtained from the flux distribution.

$$r_i = \sum_{N_{ij}>0,j} N_{ij} v_{f,j} - \sum_{N_{ij}<0,j} N_{ij} v_{b,j}$$

$$r_{M1\_c} = (1)v_{f1} + (1)v_{f2} = 1.2$$

$$r_{M1\_m} = (1)v_{f3} - (-1)v_{b5} = 1.6$$

- The overall flux-sum synthesis rates for the metabolite, M1:

$$r_m = \sum_{i\in\Omega^c} \left(\sum_{N_{ij}>0,j} N_{ij} v_{f,j} - \sum_{N_{ij}<0,j} N_{ij} v_{b,j} \right), m \in \Omega^m$$

$$r_{M1} = r_{M1\_c} + r_{M1\_m} = 2.8$$

3. Flux Patterns for Normal, Cancer and Mutants

- Suppose that a metabolic network consisted of 8 metabolites, and their synthesis rates for the normal model, cancer model and three mutant models are shown as follows:

Synthesis rate for each metabolite:

$$r_m = \sum_{m_i \in \Omega^c} \left(\sum_{N_{ij}>0,j} N_{ij} v_{f,j} - \sum_{N_{ij}<0,j} N_{ij} v_{b,j} \right), m \in \Omega^m$$

Flux Pattern					
	Normal	Cancer	Mutant 1	Mutant 2	Mutant 3
Metabolite 1	1	4	3.4822	3.249	3.321
Metabolite 2	0.5	1.4142	0.3299	1.1487	1.3193
Metabolite 3	1	0.7071	0.7579	0.8123	0.933
Metabolite 4	0.5	0.25	0.2872	1.1487	0.4353
Metabolite 5	1	1.4142	1.36	0.7579	1.0353
Metabolite 6	0	1.5	0.9	1.1	0.7
Metabolite 7	1	0	0.01	0	0
Metabolite 8	0.5	0.5	0.4999	0.5001	0.5

4. Logarithmic Fold Change Ratio for the Template and Mutants

- The logarithmic fold change ratio (LFC_m) for the template and each mutant are computed as follows:
- To avoid the numerical domain error, the logarithmic fold change ratio (LFCm) can be evaluated by

$$LFC_m^T = \log_2\left((r_{m,deficient} + 10^{-10}) / (r_{m,\text{normal}} + 10^{-10})\right)$$

$LFC_m^T = \log_2\left(r_{m,\text{cancer}} / r_{m,\text{normal}}\right)$		$LFC_m = \log_2\left(r_{m,\text{mutant}} / r_{m,\text{normal}}\right)$		
	Template	Mutant 1	Mutant 2	Mutant 3
Metabolite 1	2	1.8	1.7	1.9
Metabolite 2	1.5	-0.6	1.2	1.4
Metabolite 3	-0.5	-0.4	-0.3	-0.1
Metabolite 4	-1	-0.8	1.2	-0.2
Metabolite 5	0.5	0.45	-0.4	0.05
Metabolite 6	33.80	33.07	33.36	32.70
Metabolite 7	-33.22	-6.64	-33.22	-33.22
Metabolite 8	0	-0.2886	0.2885	0

5. Similarity Indicator and Similarity Ratio

- Suppose that we set 3% of the tolerance for increase/decrease, i.e. tol_+ = 0.0426 and tol_- = -0.0439

Similarity indicator:

$$\mu_m^T = \begin{cases} 1, \text{ if } LFC_m > tol_+ \text{ and } LFC_m^{T/T_{rxn}} > tol_+ \\ -1, \text{ if } LFC_m < tol_- \text{ and } LFC_m^{T/T_{rxn}} < tol_- \\ 0, \text{ otherwise} \end{cases}$$

Similarity ratio:

$$SR^T = \sum_{m=1}^{M} \left|\mu_m^T\right| \Big/ M$$

	Similarity indicator		
	Mutant 1	Mutant 2	Mutant 3
Metabolite 1	1	1	1
Metabolite 2	0	1	1
Metabolite 3	-1	-1	-1
Metabolite 4	-1	0	-1
Metabolite 5	1	0	1
Metabolite 6	1	1	1
Metabolite 7	-1	-1	-1
Metabolite 8	0	0	0
$SR^T = \sum_{m=1}^{8} \left\|\mu_m^T\right\| \Big/ 8$	0.75	0.625	0.875

6. Nested Hybrid Differential Evolution (NHDE)

The TLOP is formulated as in the form

$$
\begin{cases}
\text{Outer optimization problem:} \\
\max_{\delta,\mathbf{z}_i} \left(SR^{T,HT} + SR^{W,HT} \right) + SR^{T,HT} SR^{W,HT} \\
\max_{\delta,\mathbf{z}_i} \left(SR^{T,BL} + SR^{W,BL} \right) + SR^{T,BL} SR^{W,BL} \\
\text{subject to the inner optimization problems,} \\
\text{FBA problem:} \\
\begin{cases}
\max_{\mathbf{v}_{f/b}} v_{biomass} \\
\text{subject to} \\
\quad \mathbf{N}\left(\mathbf{v}_f - \mathbf{v}_b\right) = \mathbf{0} \\
\quad v_{f/b,i}^{LB,MU} \le v_{f/b,i} \le v_{f/b,i}^{UB,MU},\ z_i \in \Omega^{MU} \\
\quad v_{f/b,j}^{LB} \le v_{f/b,j} \le v_{f/b,j}^{UB},\ z_j \notin \Omega^{MU}
\end{cases} \\
\text{UFD problem:} \\
\begin{cases}
\min_{\mathbf{v}_{f/b}} \sum_{i \in \Omega^{Int}} \left(v_{f,k}\right)^2 + \left(v_{b,k}\right)^2 \\
\text{subject to} \\
\quad \mathbf{N}\left(\mathbf{v}_f - \mathbf{v}_b\right) = \mathbf{0} \\
\quad v_{f/b,i}^{LB,MU} \le v_{f/b,i} \le v_{f/b,i}^{UB,MU},\ z_i \in \Omega^{MU} \\
\quad v_{f/b,j}^{LB} \le v_{f/b,j} \le v_{f/b,j}^{UB},\ z_j \notin \Omega^{MU} \\
\quad v_{biomass} \ge v_{biomass}^{*}
\end{cases}
\end{cases}
\tag{1}
$$

$$
\begin{cases}
\text{Upregulation:} \\
\begin{cases}
\left(1-\delta\right) v_{f,i}^{basal} + \delta v_{f,i}^{UB} \le v_{f,i} \le v_{f,i}^{UB} \\
v_{b,i}^{LB} \le v_{b,i} \le \left(1-\delta\right) v_{b,i}^{basal} + \delta v_{b,i}^{LB};\ z_i \in \Omega^{MU}
\end{cases} \\
\text{Downregulation:} \\
\begin{cases}
v_{f,i}^{LB} \le v_{f,i} \le \left(1-\delta\right) v_{f,i}^{basal} + \delta v_{f,i}^{LB} \\
\left(1-\delta\right) v_{b,i}^{basal} + \delta v_{b,i}^{UB} \le v_{b,i} \le v_{b,i}^{UB};\ z_i \in \Omega^{MU}
\end{cases} \\
\text{Knockout:} \\
v_{f,i} = v_{b,i} = 0;\ z_i \in \Omega^{MU}
\end{cases}
\tag{2}
$$

Definition of symbol in Equations (1) and (2) are defined as follows:

$\mathbf{N}$	The $m \times n$ stoichiometric matrix
$SR^{T,BL/HT}$	The similarity ratios for the template of flux-sum alterations compared with CA to BL/HT models, respectively

$SR^{W,BL/HT}$ The similarity ratios for the template of flux-sum alterations compared with Warburg hypothesis to BL/HT models, respectively.

$v_{biomass}$ The biomass growth rate

$v_{f/b,i}$ The forward and backward flux of the ith reaction

$v_{f/b,j}^{LB}$ The positive lower bounds of the jth forward/backward flux

$v_{f/b,j}^{UB}$ The positive upper bounds of the jth forward/backward flux

$v_{f/b,i}^{LB,MU}$ The lower bounds of the ith upregulation, downregulation, or knockout flux due to the ith enzyme dysregulation

$v_{f/b,i}^{UB,MU}$ The upper bounds of the ith upregulation, downregulation, or knockout flux due to the ith enzyme dysregulation

z_i The integer variables zi are used to determine the dysregulated enzymes, so that the bounds of the modulated reactions are restricted to be upregulation. downregulation and knockout reactions

δ The dysregulation strength parameter to be determined within 0 and 1

Ω^{Int} The set of intracellular reactions

Ω^{MU} The set of mutated reactions

TLOP is applied to mimic mutant schemes in a wet lab for oncogene inference. The NHDE algorithm, extended from HDE, is used to identify the integer variables, z_i, and the dysregulation strength parameter, δ, of the outer optimization problem in the oncogene inference optimization problem described by Eq. (1). The decision variables are then provided for FBA and UFD problems in the inner optimization problem to hierarchically compute flux distribution for each mutant. The fluxes acted as the decision variables in FBA and UFD problems are applied for evaluating the similarity ratios in the outer problem.

The basic operations of NHDE is a parallel direct search algorithm that use the N_p vectors of the integer/real decision variables in optimization problems for the population of the generation G. They begin with a population of N_p possible solutions selected assuming a uniform probability distribution for each decision parameter vector. The computational steps of the algorithm are listed as follows:

1. Representation and initialization

$$(\mathbf{z}^0)_i = \text{uniformInt}(\mathbf{z}^{\min}, \mathbf{z}^{\max}), i = 1, \ldots, N_p$$

Each individual is generated by an integer random number between $\mathbf{z}_{\min}$ and $\mathbf{z}_{\max}$ with uniform distribution

2. Mutation with rounding operation

$$(\hat{\mathbf{z}}^G)_i = \text{INT}\left\{(\mathbf{z}^G)_p + \rho^G\left[(\mathbf{z}^G)_j - (\mathbf{z}^G)_k + (\mathbf{z}^G)_l - (\mathbf{z}^G)_m\right]\right\}$$

3. Crossover operation

$$z_{ji}^G = \begin{cases} z_{ji}^{G-1}, & \text{if a random number } > C_R \\ \hat{z}_{ji}^G, & \text{otherwise}, j = 1, \ldots, n; i = 1, \ldots, N_p \end{cases}$$

4. Restriction operation

$$z_{ji}^G = \begin{cases} z_{ji}^G, z_{ji}^G \in [z_j^{\min}, z_j^{\max}] \\ \text{uniformInt}(z_j^{\min}, z_j^{\max}), z_{ji}^G \notin [z_j^{\min}, z_j^{\max}] \end{cases}$$

5. Selection and evaluation
   (a) For each individual, solve FBA problem by a linear optimization solver to obtain its maximum biomass growth rate

$$v_{biomass}^* = \arg\left\{\begin{array}{l} \max\limits_{\mathbf{v}_{f/b}} v_{biomass} \\ \text{subject to} \\ \quad \mathbf{N}\left(\mathbf{v}_f - \mathbf{v}_b\right) = \mathbf{0} \\ \quad v_{f/b,i}^{LB,MU} \le v_{f/b,i} \le v_{f/b,i}^{UB,MU}, \; z_i \in \Omega^{MU} \\ \quad v_{f/b,j}^{LB} \le v_{f/b,j} \le v_{f/b,j}^{UB}, \; z_j \notin \Omega^{MU} \end{array}\right\}$$

   (b) For each individual and its maximum biomass growth rate, solve UFD problem by a quadratic optimization solver to obtain the optimal flux distribution

$$\left\{\begin{array}{l} \min\limits_{\mathbf{v}_{f/b}} \sum\limits_{i \in \Omega^{Int}} \left(v_{f,k}\right)^2 + \left(v_{b,k}\right)^2 \\ \text{subject to} \\ \quad \mathbf{N}\left(\mathbf{v}_f - \mathbf{v}_b\right) = \mathbf{0} \\ \quad v_{f/b,i}^{LB,MU} \le v_{f/b,i} \le v_{f/b,i}^{UB,MU}, \; z_i \in \Omega^{MU} \\ \quad v_{f/b,j}^{LB} \le v_{f/b,j} \le v_{f/b,j}^{UB}, \; z_j \notin \Omega^{MU} \\ \quad v_{biomass} \ge v_{biomass}^* \end{array}\right.$$

   (c) Compute fitness for each individual

$$f\left(z_i^G\right) = F(SR^{T/W}) + \text{penalty}$$

   (d) Apply the one-to-one competition between the parent and its offspring to

yield the next generation

$$z_i^G = \arg\max\left\{f\left(z_i^{G-1}\right), f\left(z_i^G\right)\right\}, i = 1,\ldots, N_p$$

(e) Select the best individual from the population

$$z_{best}^G = \arg\max\left\{ f\left(z_i^G\right), i = 1,\ldots, N_p\right\}$$

6. Migration operation performed naturally or enforced if necessary

$$(\mathbf{z}^G)_i = \text{uniformInt}(\mathbf{z}^{\min}, \mathbf{z}^{\max}),\ \text{if } \zeta \le \varepsilon = [0,1]$$

7. Repeat steps 2 to 6

In this study, four parameters in the NHDE algorithm require to be provided by the user as following: CR = 0.5, ε = 0.05, N_p = 50, and the maximum number of iterations = 100. ρ^G = random number between 0 and 1.

7. Categories of Flux Variances Between the Normal and Mutant Models

- The black line indicates the flux interval of the normal model, and the red line indicates that of the mutant/cancer model.
- Number in the brackets is the indicator to denote each category.

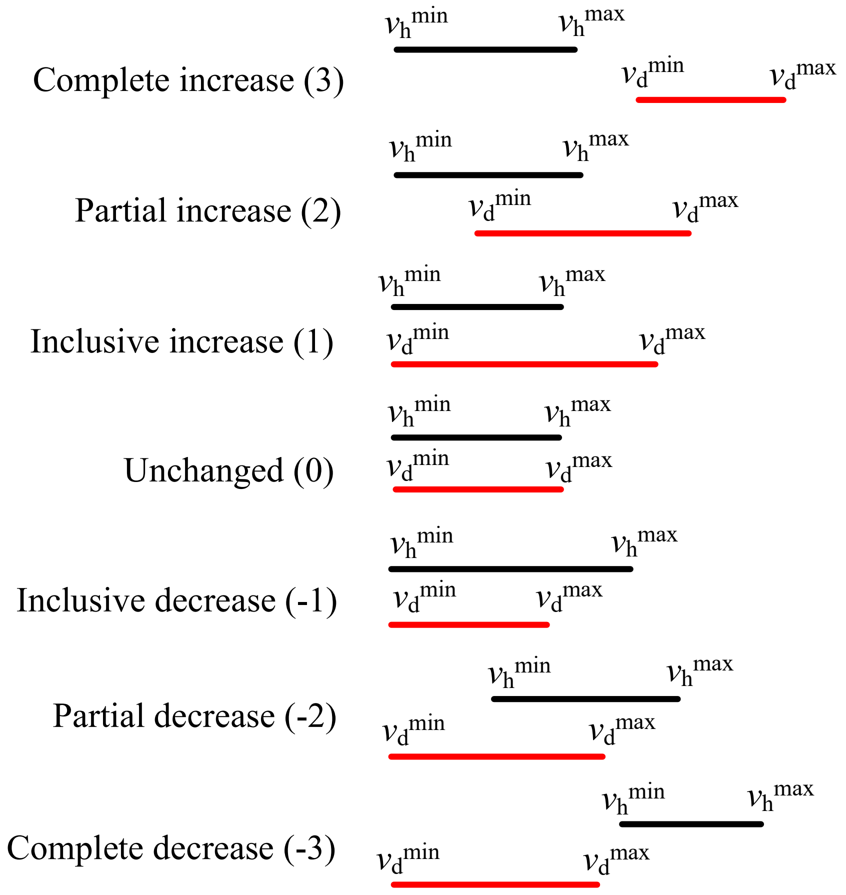

Supplement: Toy examples [file rsos191241supp1.pdf]
